# Study of curriculum of Doctor of Physical therapy programme based on World Federation of Medical Education standards

**DOI:** 10.12669/pjms.346.15926

**Published:** 2018

**Authors:** Syed Shakil-ur-Rehman, Shakeel Ahmad, Raheela Yasmin

**Affiliations:** 1 *Syed Shakil-ur-Rehman, PhD PT. Professor/Principal, Riphah College of Rehabilitation Sciences, Riphah International University, Islamabad, Pakistan*; 2 *Shakeel Ahmad, Assistant Professor, Riphah College of Rehabilitation Sciences, Riphah International University, Islamabad, Pakistan*; 3 *Raheela Yasmin, Professor, Islamic International Medical College, Riphah International University, Islamabad, Pakistan*

**Keywords:** Doctor of Physical Therapy, Program evaluation, Physical Therapy Education

## Abstract

**Objective:**

To evaluate the curriculum for Doctor of Physical Therapy (DPT) programme based on World Federation of Medical Education (WFME) standards.

**Methods:**

A questionnaire was constructed based on WFME ‘Should’ and ‘Must’ standards. It was validated by five experts in two rounds. It is comprised of Items/ questions with Yes/No options. The questionnaire was filled by the DPT Faculty and final year students at Riphah International University, Islamabad from March 01, to April 30, 2017.

**Results:**

The key weakness identified were students participation in program management, evaluation, mission statement, program designing, curriculum committee, students activities and organization, and other matters relevant to students, followed by the use of external examiners, reliability and validity of assessment tools, scrutiny of assessments by external examiners and feedback to the students on assessment. The integration of behavioral and social sciences, readiness of graduates for postgraduate studies, institutional autonomy and academic freedom for curriculum development and designing, and opportunity for the participation of other stakeholders were identified as strengths.

**Conclusions:**

As per WFME standards the curriculum for DPT program needs improvements in student’s assessments and their participation in program management, evaluation, mission statement and designing, along with facilitation in student’s activities, organizations. Strengths of the curriculum were integration of behavioral and social sciences, readiness for postgraduate studies, institutional autonomy and academic freedom for the development and designing of curriculum, and the participation of other stakeholders.

## INTRODUCTION

Programme evaluation is the key component of academic programme carried out against certain standards and criteria developed by professional institutions/organizations at international and global level.^1^-^4^ It is used to determine strength and weaknesses and provide guidance for programme analysis and Upgradation.^5^ The curriculum for DPT program was evaluated as per WFME standards in the current study.

Physical Therapy (PT) is an autonomous health care profession concerned with the promotion, restoration and maintenance of optimal physical function in population across their life span. Entry-level education in PT range from Bachelor of Sciences to doctoral level in different countries and approved by World Confederation of Physical Therapy (WCPT).^6^ The first school was established in 1955 in Karachi and started diploma in PT, upgraded to university level by awarding three years Bachelor of Sciences degree in 1973 and into four years BS program in 2000. Finally a five years DPT programme was started in 2007 at Riphah International University and currently more than 100 institutions/universities, which offering this program.^7^

Higher Education Commission (HEC) of Pakistan is the government body dealing with the accreditation and regulation of universities in the country. HEC formulated a National Curriculum revision committee (NCRC) in 2009 comprising PTs form the key universities and succeed in development of first uniform curriculum for DPT program and implemented in 2011. This curriculum was revised in 2016 and implemented in 2017 across the country.^8^ WFME developed global standards and sub standards for quality enhancement of basic education in medical disciplines; focusing nine areas and 35 sub areas. They key nine areas covered by WFME standards are mission and outcomes, educational programme, assessment of students, students, academic staff/faculty, educational resources, programme evaluation, governance and administration and Continuous renewal.^9^

## METHODS

This study was conducted at Riphah College of Rehabilitation Sciences, Islamabad, which is a constituent institution of Riphah International University, Islamabad, from March 1, to April 30, 2017. A questionnaire was developed based on WFME ‘Should’ and ‘Must’ standards and validated by five medical education experts in two rounds and finally approved. Questionnaire comprised of Items/ questions with Yes/No options.

The participants were 50 faculty members and 100 final year DPT students at Riphah College of Rehabilitation Sciences. The questionnaire was circulated and filled by the study participants in hard form. Ethical permission of the current study was taken from ethic review committee at Riphah College of Rehabilitation Sciences, Riphah International University, Islamabad. The written consent from the participant was also taken by ensuring confidentiality of data. Data was manually analyzed and strengths and weaknesses were calculated in percentage.

## RESULTS

The key weakness found were students participation in program management, evaluation, mission statement, program designing, curriculum committee, students activities and organization, and other matters relevant to students, followed by the use of external examiners, reliability and validity of assessment tools, scrutiny of assessments by external examiners and feedback to the students on assessment. The key strengths of the curriculum were integration of behavioral and social sciences, readiness of graduates for postgraduate studies, institutional autonomy and academic freedom for the development and designing of curriculum, opportunity for the participation of other stakeholders. Detailed description of key strengths and weakness results are given in Table-I.

**Table-I:** Key strengths and weakness of Curriculum for the “Doctor of Physical Therapy” Program based on World Federation of Medical Education standards.

Strengths of the Program Curriculum		Weaknesses of the Program Curriculum	
Standard /Area	% of Response “Yes”	Standard /Area	% of Response “No”

Integration of Behavioral Sciences in the curriculum	96%	Students participation in program Management	92%
Prepared and ready for post graduate medical education	95%	Students participation in Other matters relevant to students	91%
Institutional autonomy and academic freedom in the designing of the curriculum	95%	Students participation in program Evaluation	90%
Opportunity for participation to other key stakeholders	94%	Encourage the use of external examiners	90%
Integration of Social Sciences in the curriculum	93%	Student representation in mission statement	88%
Programme structure, composition and duration	92%	Student participation in Program designing	88%
Intended educational outcomes publically known	92%	Student and staff participation in curriculum committee	88%
Define administration and governance structure and its relationship with in university	92%	Evaluation/documentation of reliability and validity of the assessment tools	85%
Development of teaching and assessment methods	91%	Scrutiny of assessments by external expertise,	82%
Relationship between mission and selection of students	91%	Encouragement of student activities and organization	82%
Horizontal integration associated with disciplines and subjects	90%	Feed back to the students on assessments	79%
Vertical alignment of clinical sciences	90%	Modification of program in response to the community and society	69%
Routine curriculum monitoring of process and outcomes	90%	Interface with complementary medicine	56%

## DISCUSSION

It is vital to achieve competence for medical professionals to fulfill the community health care needs at national and international covering both developed and developing countries level. To achieve this goal the WFME global standards are one of the key guidelines for medical and allied health care schools/institutions. Therefore this study was designed to evaluate the curriculum of DPT Programme based on WFME standards, which was developed in 2005 by the World Health Organization (WHO) and WFME.^10^,^11^,^12^

The key weaknesses identified were lacking of students participation in program management, evaluation, designing and other matters relevant to students. They were less represented in mission statement and curriculum committee. Assessment of students were lacking in the use of external examiners, validity and reliability of assessment tools, scrutiny by external experts and feed back to students on assessment. There was also less encouragement found for student’s activity and organization. Modification of the program in response to the community and society was also identified a key weakness. Curriculum had fewer interfaces with complementary medicine. There are only few institutions based on quality assurance systems and external evaluation, while majority are only general criteria’s for higher education in medical and allied health professions in Pakistan.^13^

The key strength identified were the integration of behavioral and social sciences along with prepared and readiness for postgraduate PT education. There was intuitional autonomy and academic freedom found in designing the curriculum with opportunity of participation for other key stakeholders. The duration, structure and composition of the program, and the education outcomes were publically known. Administration and governance structure and its relationship with university was found well defines. Development of teaching and assessment methods, relationship between mission and selection of students, horizontal and vertical integration of curriculum with clinical sciences, and routine curriculum monitoring of processes and outcomes were identified as key strength.

There is no gold standard model for physical therapy practice, but there are systems which can provide guidelines for quality physical therapy education and practice like World Confederation of Physical Therapy (WCPT), American Physical Therapy Association (APTA) and other professional bodies at country level.^14^ The current physical therapy education is based on academic teaching/education, advanced training and clinical practice, which required quality and accredited programme as per global standards of WFME for medical and allied health professions.^15^

## CONCLUSION

It is concluded that as per the WFME standards the areas of curriculum for DPT program need improvements as regards students participation in program management, evaluation, mission statement, program designing, curriculum committee, students activities and organization. Other matters relevant to students, the use of external examiners, reliability and validity of assessment tools, scrutiny of assessments by external examiners and feedback to the students on assessment. The areas already considered satisfactory were integration of behavioral and social sciences, readiness of graduates for postgraduate studies, institutional autonomy and academic freedom for the development and designing of curriculum, opportunity for the participation of other stakeholders.

### Authors Contribution

**SSR, SA & RY**: Conceived, designed and did statistical analysis & editing of manuscript.

**SSR, RY & SA**: Did data collection and manuscript writing.

**SSR** Finally approved the article.

**SSR:** Takes the responsibility and is accountable for all aspects of the work in ensuring that questions related to the accuracy or integrity of any part of the work are appropriately investigated and resolved.
